# Lingual botryomycoma in the aftermath of Stevens-Johnson syndrome

**DOI:** 10.11604/pamj.2015.21.35.7036

**Published:** 2015-05-18

**Authors:** Houyam Moundib, Fouzia Hali

**Affiliations:** 1 Department of Dermatology and Venereology, University Hospital Ibn Rochd, Casablanca, Morocco

**Keywords:** Granulation tissue, botryomy coma, tongue

## Image in medicine

A 55 years old woman, was hospitalized in November 2011 for a Stevens-Johnson syndrome with severe mucosal impairment appeared three weeks after taking allopurinol (Zyloric^®^) for an hyperuricemia associated with arthralgia. She was put under symptomatic treatment after discontinuation of the offending molecule with a good mucocutaneous improvement. After healing of lesions of the oral mucosa in December 2011, the examination of the oral cavity has found a nodular median lesion of the dorsal surface of the tongue, in favor of a granulation tissue at the histology with spontaneous regression of the rest of lesion. The current decline is two years and two months without local recurrence.

**Figure 1:**
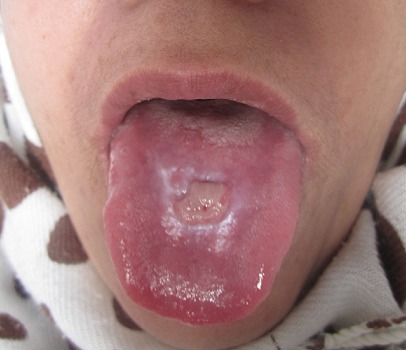
A nodular median lesion of the dorsal surface of the tongue

